# 2370: Understanding epicardial fat biology by imaging

**DOI:** 10.1017/cts.2017.225

**Published:** 2018-05-10

**Authors:** Jadranka Stojanovska, Thomas Chenevert, Alex Tsodikov, Carey Lumeng, Charles Burant

**Affiliations:** University of Michigan School of Medicine, Ann Arbor, MI, USA

## Abstract

OBJECTIVES/SPECIFIC AIMS: The goal is to understand the underlying mechanism of epicaridial fat biology and its response to cardiometabolic disease by using quantitative multi-echo Dixon (mDixon) of water and lipid sequence, T2* blood-oxygen-level-dependent (BOLD) sequence of iron content, and data analysis methods to determine the quantity of brown Versus white fat. To accomplish this goal, we propose to define the histological, genetic, and metabolite state of epicardial fat and to confirm the relationship between fat phenotype and magnetic resonance (MR) characteristics. We will then investigate whether MR is more effective in identifying patients with lower cardiovascular disease risk than computed tomography (CT). METHODS/STUDY POPULATION: We will recruit 100 patients undergoing open-heart surgery and will quantify mDixon (proton density fat fraction), BOLD (T2*), and T2/T1 maps of epicardial, extrapericardial, and subcutaneous fat before their surgery. We will then (a) validate MR findings by direct depot-specific tissue analysis for lipid content, histological, and genetic markers of inflammation and brown and white fat, (b) develop plasma and fat depot specific metabolite profiling of cardiovascular disease risk and correlate with imaging characteristics. We will categorize cardiovascular risk score (Cardiovascular Health Status) of our 100 patients on quartiles. We will then build models where the categorized cardiovascular risk score are regressed on MR measures (epicardial fat fraction, T2*, and T2/T1 maps) and CT measures (epicardial fat volume and coronary calcium score). RESULTS/ANTICIPATED RESULTS: We anticipate to learn about epicardial fat biology and the role of inflammation in cardiometabolic disease. We will validate proton density fat fraction, T2* and T2 map against histology of epicardial fat for lipid content, established markers of brown and white fat and inflammation, respectively, to help us translate imaging technique to clinical practice. In respect to our second aim we anticipate that MR identifies patients at lower cardiovascular risk quartile than CT. DISCUSSION/SIGNIFICANCE OF IMPACT: Interest in epicardial fat as a visceral fat of the heart and coronary arteries is rapidly growing as the scientific based evidence indicates that the anatomic specificity is an important contributor to the cardiovascular diseases. The transformation of epicardial fat from a cardioprotective phenotype to a pro-inflammatory, atherosclerosis-promoting state triggers inflammation that is coincident with the expansion of epicardial fat volume detected by anatomic imaging. This study will impact the management of patients at risk for cardiovascular disease because it will demonstrate that quantification of epicardial fat status by MR identifies fat tissue changes validated by histology at lower cardiovascular disease risk quartile than CT.

